# COVID-19 and SARS-CoV-2 Vaccines: A Cameo Role for Headache

**DOI:** 10.3390/ijerph20053914

**Published:** 2023-02-22

**Authors:** Paolo Martelletti

**Affiliations:** Department of Clinical and Molecular Medicine, Sapienza University, 00189 Roma, Italy; paolo.martelletti@uniroma1.it

**Keywords:** headache, migraine, SARS-CoV-2, COVID-19, vaccines, cerebral venous thrombosis, thrombotic complications

## Abstract

Headache is a very frequent symptom in COVID-19 and SARS-CoV-2 vaccination. Many studies have emphasized its clinical diagnostic and prognostic importance on the one hand, as in many cases these aspects have been completely ignored. It is therefore opportune to go back over these lines of research in order to gather what usefulness the headache symptom may or may not represent for the clinician dealing with COVID-19 or performing or following up on the clinical course following vaccination for SARS-CoV-2. The clinical evaluation of headache in COVID-19 is not fundamental in the diagnostic and prognostic process of the emergency departments; however, the risk of severe adverse events, although very rare, must be taken into account by the clinicians. For subjects presenting with severe, drug-resistant, and delayed-onset post-vaccination headache, it could represent a possible sign of central venous thrombosis or other thrombotic complications. Thus, a re-reading of the role of headache in COVID-19 and SARS-CoV-2 vaccination seems clinically useful.

## 1. Introduction

Three years after the outbreak of the COVID-19 pandemic, we can pinpoint that vaccination campaigns for SARS-CoV-2 together with stringent public policies have helped to contain the pandemic, which up to the time of writing, has caused 651 million cases of COVID-19 and 6.7 million deaths, and has resulted in the administration of 13 billion doses of vaccine [1]. Certainly, new outbreaks and/or possible pandemic resurgences may still develop, as seems to be the case in the country of origin of the pandemic, despite showing very low official figures of COVID-19 (62,723 cases and 7 deaths) in the November 2022 China Centre of Diseases Control report [2]. The importance of variants of concern (VOC) now sees the Omicron form with its six variants expanding [3], and this may imply an increase in transmissibility, virulence, and change in clinical features. Consequently, the health alert levels of prevention, diagnosis, therapy, and vaccination still need to be maintained.

In this context, this paper will present a description of the role of a very frequent COVID-19 symptom, which is observed in about one quarter of affected individuals: headache [4]. What follows will be a description of the clinical diagnostic and prognostic aspects of headache regarding COVID-19 disease and also its role in vaccinated individuals.

## 2. COVID-19 and Headache

The consideration of headache as a clinically relevant symptom of COVID-19, as well as a possible sign of mild adverse events in the immediate post-vaccinal phase or as a serious warning sign for serious adverse events due to vaccination, has been the subject of clinical reports and systematic reviews, including the persistence in long COVID headache [5,6,7,8,9]. Regarding the last point, the current recommendation is to assign a final value to headache in multisystem COVID-19 and in mild SARS-CoV-2 infection. Additionally, given that SARS-CoV-2 carries a high incidence of ageusia and anosmia, other clinical symptoms such as headache could be scaled up in priority in terms of diagnostic importance. This is what a copious amount of literature has largely reported in all clinical areas involved with SARS-CoV-2 infection. Indeed, as far as headache in COVID-19 is concerned, it is surprising to see retrospectively both how much has been published and what has been published, even though the scientific community was going through a period of great uncertainty due to a lack of knowledge and consequently a great surrounding scientific fervor. At the time of writing, a search on PubMed using the MeSH term “COVID-19” generates about 327,000 results; if we then add the term “headache”, we get about 3000 results—about 0.9% as many results. The number of results can be few if we refer to a multi-system disease that is a clinical mutant and with a trend toward the reduction of secondary transmission and immune evasion. Furthermore, this non-extensive scientific interest within the torrent of scientific publications during the pandemic raises some questions [10].

The clinical use of the headache symptom in the clinical/therapeutic course has always been of modest usefulness [11] in emergency departments, intensive care units, as well as low and medium settings, such as the wards of the internal and emergency medicine departments, as it does not represent a main alert sign for the risk of death, even though it has previously been observed in 1684 out of 3044 hospitalized patients [12]. Therefore, it can now be affirmed that any doubts about the relevance of research on COVID-19-related headache, apart from the transient clinical worsening observed with primary headaches, especially migraine without aura, can be definitively dismissed [13].

## 3. SARS-CoV-2 Vaccines and Headache

A recent meta-analysis on the occurrence of headache as an AE after vaccination for SARS-CoV-2 showed that headache is the third most common AE associated with SARS-CoV-2 vaccination, occurring in 22% of individuals after the first vaccination dose and 29% of individuals after the second dose over a 7-day period; these rates are higher when compared with those who received placebo (10–12%) and to population studies showing a 6–17% probability of individuals having a headache on the previous day [7]. There were no significant variations in the frequency of headache observed with the different vaccine types. In about one-third of cases, the features of this resolving headache are like those of migraine with pulsating quality, phono, and photophobia, while exacerbation with physical activity is more frequent (40–60% of cases). The onset is mostly within the first 24 h, and many patients have been on some form of medication to treat the headache, with acetylsalicylic acid considered to be the most efficacious [7].

If we then consider the impact on migraine of vaccinations, a recent study showed that out of 841 migraine patients who filled out an online questionnaire, 66.47% and 60.15% of patients experienced a migraine(-like) attack that occurred within 1 h to 7 days of their first and second vaccination, respectively [14]. It is, however, important to note that 57.60% of these patients reported these attacks to be more severe (50.62% of patients), longer lasting (52.80%), or resistant (49.69%) than their other case of migraine attacks. The type of causation between the two events (specific/inflammatory or non-specific/triggering) has not yet been clarified, although the immediate temporal relationship is clear [14].

Conversely the risk of serious AE is inherent in post-vaccination headaches with delayed presentation (±7 days), which may represent a red flag for secondary headaches due to cerebral venous thrombosis (CVT) and other thrombotic complications [9]. The symptomatology starts about 7 days after vaccination; the headache lacks its typical characteristics and is progressively worse, unresponsive to common analgesics, and insidious, especially in migraine sufferers who underestimate the difference compared to their usual migraine crises, resulting in delayed consultation, and potentially leading to a late-onset CVT diagnosis.

## 4. Conclusions

Vaccination for SARS-CoV-2 has played a key role in the pandemic in reducing viral aggressiveness and the subsequent clinical manifestations of COVID-19. The emergence of SARS-CoV-2 VOC with an increased tropism for the upper respiratory tract has favored a reversal of its life-threatening risk.

In the light of the scientific evidence accumulated during these last three years of the pandemic, it can be affirmed that headache has not had either a diagnostic or a prognostic role in COVID-19; however, it may be present for a long time in the consortium of symptoms of long-COVID. Of considerable importance, on the other hand, is delayed headache after vaccination for SARS-CoV-2, which may underlie an elevated risk of CVT and/or other thrombotic complications and should therefore be carefully looked for in the case of post-vaccination headache occurring around seven days after vaccination. However, early post-vaccination headache does not pose any level of risk, other than that in subjects already suffering from migraine of triggering an attack that is more severe, longer, and refractory to common acute treatment than usual [15].

Among children and adolescents infected by SARS-CoV-2, a high rate of asymptomatic carriage has been observed [16], and the safety of current COVID-19 vaccines has remained acceptable [17].

Thus, in terms of considering an overall framework for the headache during COVID-19, or immediately after vaccination for SARS-CoV-2 in both migraine and non-migraine subjects, the role of headache contextualized in a broader holistic view of medicine can rightly be described as a cameo. However, the attention that needs to be paid to the rare forms of late post-vaccination thrombotic risks is quite different [18].
